# Crystal structure of {(*S*)-1-phenyl-*N*,*N*-bis­[(pyridin-2-yl)meth­yl]ethanamine-κ^3^ *N*,*N*′,*N*′′}bis­(thio­cyanato-κ*N*)zinc from synchrotron data

**DOI:** 10.1107/S2056989016019253

**Published:** 2017-01-01

**Authors:** Dong Won Lee, Jong Won Shin

**Affiliations:** a Daegu-Gyeongbuk Branch, Korea Institute of Science and Technology Information, 90 Yutongdanji-ro, Buk-gu, Daegu 41515, Republic of Korea

**Keywords:** crystal structure, chiral ligand, sodium thio­cyanate, π–π inter­actions, synchrotron data

## Abstract

The title Zn^II^ complex, [Zn(NCS)_2_(C_20_H_21_N_3_)], has been characterized by synchrotron single-crystal diffraction and FT–IR spectroscopy. The central Zn^II^ ion has a distorted square-pyramidal coordination geometry, with three N atoms of the chiral (*S*) 1-phenyl-*N*,*N*-bis­[(pyridin-2-yl)meth­yl]ethanamine (*S*-ppme) ligand and one N atom of a thio­cyanate anion in the equatorial plane, and one N atom of another thio­cyanate anion at the apical position. The average Zn—N_*S*-ppme_ and Zn—N_NCS_ bond lengths are 2.183 (2) and 1.986 (2) Å, respectively. In the crystal, inter­molecular C—H⋯S hydrogen bonds and a face-to-face π–π inter­action [centroid–centroid distance = 3.482 (1) Å] link the mol­ecules and give rise to a supra­molecular sheet structure parallel to the *ac* plane.

## Abstract 2

The Zn^II^ ion in the title compound shows a distorted square-pyramidal coordination geometry with three N atoms of the chiral *S*-ppme ligand and two N atoms of the thio­cyanate anions. In the crystal, mol­ecules are connected by hydrogen bonds and π–π inter­actions, forming a two-dimensional supra­molecular network parallel to the *bc* plane.

## Chemical context   

Recently, the preparation of new polyamines or their derivatives have attracted increasing attention in organic chemistry, pharmaceutical chemistry and materials science because they can easily inter­act with metal ions and form stable multifunctional compounds with various applications in magnetic materials, sorption materials, as well as fluorescent substances (Lodeiro & Pina, 2009 ▸; Nowicka *et al.*, 2011 ▸; Yao *et al.*, 2015 ▸). For instance, metal complexes with cyclam or aza­macrocyclic ligands have been synthesized and investigated for selective adsorption of CO_2_ over N_2_ gases (Huang *et al.*, 2013 ▸). In particular, chiral derivatives based on polyamine ligands can easily form chiral metal complexes with inter­esting properties, such as chiral recognition or as asymmetric catalysts. For example, the chiral two-dimensional coordination polymer, [Ni(*L* ^*R*,*R*^)]_3_[C_6_H_3_(COO)_3_]_2_·12H_2_O·CH_3_CN {*L* ^*R*,*R*^ is 1,8-bis[(*R*)-α-methyl­benz­yl]-1,3,6,8,10,13-hexa­aza­cyclo­tetra­deca­ne}, showed an efficient chiral recognition for *rac*-1,1′-bi-2-naphthol (Ryoo *et al.*, 2010 ▸). Moreover, a chiral iron(III) complex containing binol derivatives exhibited high enanti­o­selectivity and high yield for the enanti­opure β-amino alcohols (Tak *et al.*, 2016 ▸). Nevertheless, only a few of these complexes have been reported and characterized because the preparation of these complexes remains a major challenge in synthetic chemistry and materials science (Gu *et al.*, 2016 ▸). The thio­cyanate ion is a versatile anion which can bridge to metal ions through the S or N atom, thus allowing the assembly of supra­molecular compounds (Nawrot *et al.*, 2016 ▸). We report here the preparation and crystal structure of a chiral zinc complex constructed from the versatile tridentate chiral ligand (*S*)-1-phenyl-*N*,*N*-bis­[(pyridin-2-yl)meth­yl]ethanamine (*S*-ppme) and the thio­cyanate ion, namely [Zn(NCS)_2_(*S*-ppme)].
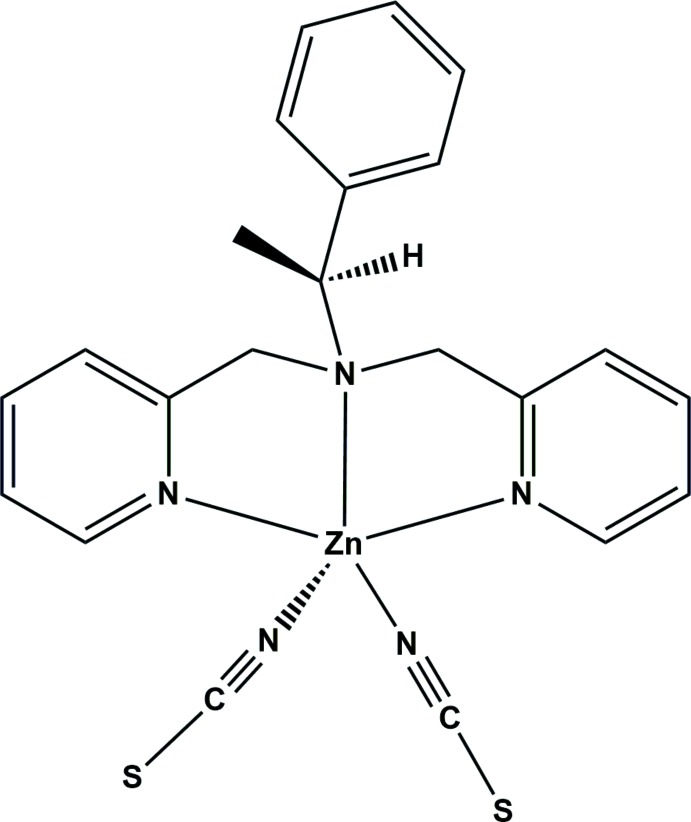


## Structural commentary   

**Figure 1:**
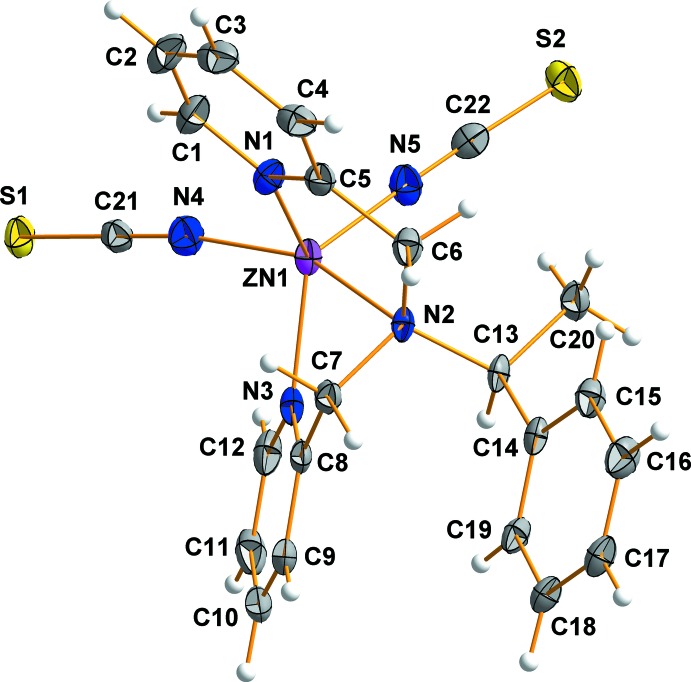
A view of the mol­ecular structure of the title compound, showing the atom-labelling scheme, with displacement ellipsoids drawn at the 50% probability.

A view of the mol­ecular structure of the title compound is shown in Fig. 1 ▸. The coordination environment of the Zn^II^ ion can be described as distorted square pyramidal. The Zn^II^ ion is coordinated by three N atoms from the chiral *S*-ppme ligand and by two N atoms of thio­cyanate ions. The thio­cyanate ions coordinate through the N atoms in *cis* positions with respect to each other and are *trans* to the phenyl group of the chiral *S*-ppme ligand. The coordinating thio­cyanate ions are linear but slightly bent in relation to the Zn^II^ ion [N4—C21—S1 = 179.9 (1)°, N5—C22—S2 = 178.5 (4)°, Zn1—N4—C21 = 171.6 (4)° and Zn1—N5—C22 = 170.3 (4)°]. The bond angle between the thio­cyanate ions is 101.43 (2)°. The average N≡C and C—S bond lengths of the thio­cyanate ions are 1.158 (4) and 1.629 (6) Å, respectively, which implies that both thio­cyanate ions are not delocalized. The former is very similar to the C≡N triple-bond length, while the latter is slightly shorter than reported C—S single-bond length (Hashem *et al.*, 2014 ▸). The pyridine rings of the *S*-ppme ligand are twisted with respect to each other. The average Zn—N_*S*-ppme_ and Zn—N_NCS_ bond lengths are 2.183 (2) and 1.986 (2) Å, respectively. The bond angles around the Zn^II^ ion range from 73.99 (1) to 156.01 (1)°.

## Supra­molecular features   

**Figure 2:**
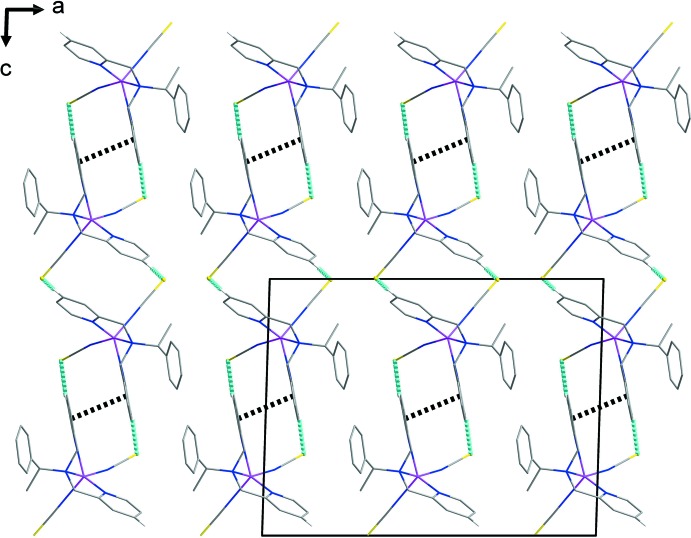
A view of the crystal-packing structure for the title compound, showing the C—H⋯S hydrogen bonds (sky-blue dashed lines) and π–π inter­actions (black dashed lines).

The thio­cyanate ligands form inter­molecular C—H⋯S hydrogen bonds with adjacent pyridine groups of the chiral *S*-ppme ligand, giving rise to a sheet structure parallel to the *ac* plane (Fig. 2 ▸ and Table 1 ▸) (Steed & Atwood, 2009 ▸). In the sheet, adjacent C8–C12/N3 pyridine rings of chiral *S*-ppme ligands are also linked through a face-to-face π–π inter­action, with a centroid–centroid distance of 3.482 (1) Å and a dihedral angle of 2.947 (1)°.

## Database survey   

A search of the Cambridge Structural Database (Version 5.37, February 2016 with two updates; Groom *et al.*, 2016 ▸) gives three copper(II) complexes with the same chiral *S*-ppme ligand (Rowthu *et al.*, 2011 ▸; Woo *et al.*, 2011 ▸) for which syntheses, magnetic properties and crystal structures have been reported.

## Synthesis and crystallization   

The chiral *S*-ppme ligand was prepared according to a slight modification of the method of Rowthu *et al.* (2011 ▸). A methanol solution (5 mL) of KNCS (0.078 g, 0.80 mmol) was added slowly to a methanol solution (15 mL) containing ZnSO_4_·7H_2_O (0.115 g, 0.40 mmol). The mixture was stirred for 20 min and the the formed white precipitates were eliminated by filtration. A solution of the chiral *S*-ppme (0.121 g, 0.40 mmol) in MeOH (10 mL) was added slowly to the filtered solution with vigorous stirring at room temperature. The resulting pale-yellow precipitates were collected by filtration, washed with methanol and diethyl ether, and dried in air. Single crystals were obtained by slow evaporation from methanol solution for a period of several days (yield: 0.123 g, 64%). FT–IR (KBr, cm^−1^): 3102, 3029, 2995, 2910, 2056, 1606.

## Refinement   

Crystal data, data collection and structure refinement details are summarized in Table 2 ▸. All H atoms were placed in geometrically idealized positions and constrained to ride on their parent atoms, with C—H distances of 0.95–0.99 Å and *U*_iso_(H) values of 1.2 or 1.5*U*_eq_ of the parent atoms.

## Supplementary Material

Crystal structure: contains datablock(s) I. DOI: 10.1107/S2056989016019253/is5466sup1.cif

Structure factors: contains datablock(s) I. DOI: 10.1107/S2056989016019253/is5466Isup2.hkl

CCDC reference: 1520395

Additional supporting information: crystallographic information; 3D view; checkCIF report

## Figures and Tables

**Table 1:** Hydrogen-bond geometry (Å, °)

*D*—H⋯*A*	*D*—H	H⋯*A*	*D*⋯*A*	*D*—H⋯*A*
C3—H3⋯S2^i^	0.95	2.77	3.604 (5)	147
C11—H11⋯S1^ii^	0.95	2.80	3.738 (5)	169

Symmetry codes: (i) 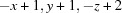; (ii) 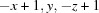.

**Table 2:** Experimental details

Crystal data	
Chemical formula	[Zn(NCS)_2_(C_20_H_21_N_3_)]
*M*_r_	484.93
Crystal system, space group	Monoclinic, *C*2
Temperature (K)	100
*a*, *b*, *c* (Å)	19.270 (4), 7.7950 (16), 14.834 (3)
β (°)	91.71 (3)
*V* (Å^3^)	2227.2 (8)
*Z*	4
Radiation type	Synchrotron, λ = 0.630 Å
μ (mm^−1^)	0.94
Crystal size (mm)	0.10 × 0.04 × 0.02

Data collection	
Diffractometer	ADSC Q210 CCD area detector
Absorption correction	Empirical (using intensity measurements) (*HKL3000sm *SCALEPACK**; Otwinowski & Minor, 1997 ▸)
*T*_min_, *T*_max_	0.912, 0.981
No. of measured, independent and observed [*I* > 2σ(*I*)] reflections	11189, 6035, 5123
*R*_int_	0.048
(sin θ/λ)_max_ (Å^−1^)	0.696

Refinement	
*R*[*F* ^2^ > 2σ(*F* ^2^)], *wR*(*F* ^2^), *S*	0.039, 0.093, 0.99
No. of reflections	6035
No. of parameters	272
No. of restraints	1
H-atom treatment	H-atom parameters constrained
Δρ_max_, Δρ_min_ (e Å^−3^)	0.35, −1.03
Absolute structure	Flack *x* determined using 2026 quotients [(*I* ^+^)−(*I* ^−^)]/[(*I* ^+^)+(*I* ^−^)] (Parsons *et al.*, 2013 ▸)
Absolute structure parameter	−0.010 (6)

Computer programs: *PAL BL2D-SMDC* (Shin *et al.*, 2016 ▸), *HKL3000sm* (Otwinowski & Minor, 1997 ▸), *SHELXS2014* (Sheldrick, 2008 ▸), *SHELXL2014* (Sheldrick, 2015 ▸), *DIAMOND* (Putz & Brandenburg, 2014 ▸) and *publCIF* (Westrip, 2010 ▸).

## supplementary crystallographic information

### Crystal data

**Table:**

[Zn(NCS)_2_(C_20_H_21_N_3_)]	*F*(000) = 1000
*M**_r_* = 484.93	*D*_x_ = 1.446 Mg m^−^^3^
Monoclinic, *C*2	Synchrotron radiation, λ = 0.630 Å
*a* = 19.270 (4) Å	Cell parameters from 32924 reflections
*b* = 7.7950 (16) Å	θ = 0.4–33.6°
*c* = 14.834 (3) Å	µ = 0.94 mm^−^^1^
β = 91.71 (3)°	*T* = 100 K
*V* = 2227.2 (8) Å^3^	Needle, colorless
*Z* = 4	0.10 × 0.04 × 0.02 mm

### Data collection

**Table:**

ADSC Q210 CCD area detector diffractometer	5123 reflections with *I* > 2σ(*I*)
Radiation source: PLSII 2D bending magnet	*R*_int_ = 0.048
ω scan	θ_max_ = 26.0°, θ_min_ = 2.4°
Absorption correction: empirical (using intensity measurements) (*HKL3000sm SCALEPACK*; Otwinowski & Minor, 1997)	*h* = −26→26
*T*_min_ = 0.912, *T*_max_ = 0.981	*k* = −10→10
11189 measured reflections	*l* = −20→20
6035 independent reflections	

### Refinement

**Table:**

Refinement on *F*^2^	Hydrogen site location: inferred from neighbouring sites
Least-squares matrix: full	H-atom parameters constrained
*R*[*F*^2^ > 2σ(*F*^2^)] = 0.039	*w* = 1/[σ^2^(*F*_o_^2^) + (0.0509*P*)^2^] where *P* = (*F*_o_^2^ + 2*F*_c_^2^)/3
*wR*(*F*^2^) = 0.093	(Δ/σ)_max_ < 0.001
*S* = 0.99	Δρ_max_ = 0.35 e Å^−^^3^
6035 reflections	Δρ_min_ = −1.03 e Å^−^^3^
272 parameters	Absolute structure: Flack *x* determined using 2026 quotients [(I+)-(I-)]/[(I+)+(I-)] (Parsons *et al.*, 2013)
1 restraint	Absolute structure parameter: −0.010 (6)

### Special details

**Table:**

Geometry. All esds (except the esd in the dihedral angle between two l.s. planes) are estimated using the full covariance matrix. The cell esds are taken into account individually in the estimation of esds in distances, angles and torsion angles; correlations between esds in cell parameters are only used when they are defined by crystal symmetry. An approximate (isotropic) treatment of cell esds is used for estimating esds involving l.s. planes.

### Fractional atomic coordinates and isotropic or equivalent isotropic displacement parameters (Å^2^)

**Table:**

	*x*	*y*	*z*	*U*_iso_*/*U*_eq_	
Zn1	0.46128 (2)	0.33954 (6)	0.76988 (3)	0.01920 (11)	
N1	0.52691 (16)	0.5093 (4)	0.8331 (2)	0.0213 (7)	
N2	0.40589 (15)	0.6204 (4)	0.7503 (2)	0.0165 (7)	
N3	0.43224 (14)	0.3562 (5)	0.6353 (2)	0.0199 (6)	
C1	0.5905 (2)	0.4552 (6)	0.8617 (3)	0.0287 (9)	
H1	0.6043	0.3414	0.8480	0.034*	
C2	0.6357 (2)	0.5585 (7)	0.9097 (3)	0.0331 (10)	
H2	0.6797	0.5161	0.9298	0.040*	
C3	0.6165 (2)	0.7254 (7)	0.9286 (3)	0.0306 (10)	
H3	0.6474	0.7999	0.9606	0.037*	
C4	0.5509 (2)	0.7824 (6)	0.8998 (3)	0.0254 (9)	
H4	0.5362	0.8960	0.9126	0.030*	
C5	0.50773 (19)	0.6714 (6)	0.8525 (3)	0.0198 (8)	
C6	0.4348 (2)	0.7272 (5)	0.8232 (3)	0.0232 (8)	
H6A	0.4042	0.7208	0.8755	0.028*	
H6B	0.4360	0.8480	0.8028	0.028*	
C7	0.44001 (19)	0.6585 (6)	0.6647 (3)	0.0194 (7)	
H7A	0.4905	0.6735	0.6761	0.023*	
H7B	0.4212	0.7666	0.6388	0.023*	
C8	0.42740 (19)	0.5140 (5)	0.5994 (3)	0.0188 (8)	
C9	0.4133 (2)	0.5404 (7)	0.5076 (3)	0.0277 (10)	
H9	0.4107	0.6529	0.4831	0.033*	
C10	0.4032 (2)	0.3974 (7)	0.4533 (3)	0.0360 (13)	
H10	0.3938	0.4108	0.3905	0.043*	
C11	0.4069 (2)	0.2344 (7)	0.4910 (3)	0.0360 (13)	
H11	0.3991	0.1356	0.4546	0.043*	
C12	0.4220 (2)	0.2182 (6)	0.5822 (3)	0.0282 (10)	
H12	0.4252	0.1069	0.6081	0.034*	
C13	0.32788 (18)	0.6288 (5)	0.7383 (3)	0.0188 (8)	
H13	0.3149	0.5409	0.6917	0.023*	
C14	0.30213 (18)	0.8003 (5)	0.7011 (3)	0.0184 (8)	
C15	0.2898 (2)	0.9419 (5)	0.7561 (3)	0.0238 (8)	
H15	0.2977	0.9328	0.8194	0.029*	
C16	0.2663 (2)	1.0954 (6)	0.7194 (3)	0.0286 (10)	
H16	0.2576	1.1899	0.7579	0.034*	
C17	0.2554 (2)	1.1123 (5)	0.6272 (3)	0.0258 (9)	
H17	0.2394	1.2179	0.6024	0.031*	
C18	0.2679 (2)	0.9745 (6)	0.5717 (3)	0.0272 (9)	
H18	0.2616	0.9858	0.5083	0.033*	
C19	0.28976 (18)	0.8195 (6)	0.6087 (3)	0.0221 (8)	
H19	0.2964	0.7241	0.5701	0.027*	
C20	0.2913 (2)	0.5759 (6)	0.8239 (3)	0.0253 (9)	
H20A	0.2982	0.6648	0.8700	0.038*	
H20B	0.3107	0.4672	0.8462	0.038*	
H20C	0.2415	0.5619	0.8104	0.038*	
N4	0.53102 (18)	0.1463 (5)	0.7434 (3)	0.0299 (8)	
C21	0.5672 (2)	0.0390 (5)	0.7176 (3)	0.0210 (8)	
S1	0.61788 (5)	−0.11203 (13)	0.68120 (7)	0.0269 (2)	
N5	0.40467 (18)	0.2318 (5)	0.8601 (3)	0.0267 (8)	
S2	0.31745 (6)	0.13587 (16)	0.99713 (8)	0.0303 (3)	
C22	0.3691 (2)	0.1913 (5)	0.9177 (3)	0.0220 (8)	

### Atomic displacement parameters (Å^2^)

**Table:**

	*U*^11^	*U*^22^	*U*^33^	*U*^12^	*U*^13^	*U*^23^
Zn1	0.01633 (18)	0.0166 (2)	0.0249 (2)	0.0000 (2)	0.00448 (14)	0.0002 (2)
N1	0.0170 (15)	0.0190 (17)	0.0276 (17)	0.0002 (14)	−0.0009 (13)	0.0016 (13)
N2	0.0123 (13)	0.0154 (16)	0.0221 (16)	−0.0017 (12)	0.0027 (12)	−0.0035 (12)
N3	0.0122 (12)	0.0209 (17)	0.0270 (15)	0.0012 (16)	0.0053 (11)	−0.0060 (15)
C1	0.0173 (18)	0.029 (2)	0.039 (2)	0.0041 (18)	−0.0019 (17)	0.0042 (19)
C2	0.0173 (19)	0.039 (3)	0.043 (3)	−0.003 (2)	−0.0062 (18)	0.004 (2)
C3	0.027 (2)	0.040 (3)	0.025 (2)	−0.012 (2)	−0.0027 (17)	0.0042 (19)
C4	0.0291 (19)	0.026 (2)	0.0212 (19)	−0.0066 (18)	0.0015 (15)	0.0004 (15)
C5	0.0170 (17)	0.021 (2)	0.0212 (18)	−0.0035 (17)	0.0021 (14)	0.0012 (15)
C6	0.0206 (18)	0.022 (2)	0.027 (2)	0.0001 (17)	0.0027 (15)	−0.0061 (16)
C7	0.0145 (16)	0.0213 (19)	0.0226 (18)	0.0009 (16)	0.0041 (14)	0.0029 (16)
C8	0.0103 (16)	0.025 (2)	0.0217 (19)	0.0004 (16)	0.0050 (14)	−0.0006 (16)
C9	0.0163 (18)	0.044 (3)	0.023 (2)	0.005 (2)	0.0043 (15)	0.0002 (19)
C10	0.0156 (17)	0.069 (4)	0.024 (2)	0.001 (2)	0.0044 (15)	−0.011 (2)
C11	0.020 (2)	0.053 (4)	0.036 (3)	−0.004 (2)	0.0055 (19)	−0.026 (2)
C12	0.018 (2)	0.027 (2)	0.040 (3)	0.0004 (19)	0.0066 (17)	−0.009 (2)
C13	0.0138 (16)	0.0162 (19)	0.027 (2)	0.0018 (15)	0.0036 (14)	−0.0021 (15)
C14	0.0110 (14)	0.016 (2)	0.0280 (19)	0.0010 (14)	0.0021 (13)	−0.0027 (14)
C15	0.0221 (19)	0.021 (2)	0.029 (2)	0.0023 (18)	0.0022 (15)	−0.0063 (17)
C16	0.026 (2)	0.023 (2)	0.037 (2)	0.0081 (19)	−0.0006 (18)	−0.0068 (18)
C17	0.0190 (19)	0.021 (2)	0.037 (2)	0.0048 (17)	−0.0008 (17)	0.0020 (17)
C18	0.0201 (19)	0.031 (2)	0.030 (2)	0.0104 (19)	−0.0016 (16)	0.0013 (18)
C19	0.0171 (15)	0.021 (2)	0.0277 (18)	0.0029 (18)	−0.0016 (13)	−0.0064 (18)
C20	0.0179 (17)	0.027 (2)	0.031 (2)	0.0028 (18)	0.0079 (15)	0.0043 (18)
N4	0.0291 (18)	0.027 (2)	0.034 (2)	0.0080 (17)	0.0060 (16)	0.0060 (16)
C21	0.0214 (18)	0.020 (2)	0.0216 (18)	0.0018 (17)	0.0034 (14)	0.0026 (15)
S1	0.0247 (5)	0.0236 (6)	0.0326 (5)	0.0079 (4)	0.0072 (4)	0.0012 (4)
N5	0.0260 (18)	0.0229 (18)	0.0316 (19)	−0.0019 (15)	0.0071 (14)	0.0042 (15)
S2	0.0287 (5)	0.0326 (6)	0.0300 (6)	−0.0088 (5)	0.0087 (4)	0.0029 (5)
C22	0.0219 (18)	0.0156 (19)	0.028 (2)	−0.0021 (17)	−0.0032 (15)	0.0011 (16)

### Geometric parameters (Å, º)

**Table:**

Zn1—N5	1.942 (3)	C9—H9	0.9500
Zn1—N1	2.039 (3)	C10—C11	1.389 (8)
Zn1—N3	2.061 (3)	C10—H10	0.9500
Zn1—N4	2.064 (4)	C11—C12	1.381 (7)
Zn1—N2	2.449 (3)	C11—H11	0.9500
N1—C5	1.350 (5)	C12—H12	0.9500
N1—C1	1.352 (5)	C13—C14	1.524 (5)
N2—C6	1.461 (5)	C13—C20	1.526 (5)
N2—C7	1.478 (5)	C13—H13	1.0000
N2—C13	1.510 (5)	C14—C19	1.392 (5)
N3—C8	1.342 (6)	C14—C15	1.397 (5)
N3—C12	1.344 (6)	C15—C16	1.385 (6)
C1—C2	1.370 (7)	C15—H15	0.9500
C1—H1	0.9500	C16—C17	1.384 (6)
C2—C3	1.383 (7)	C16—H16	0.9500
C2—H2	0.9500	C17—C18	1.378 (6)
C3—C4	1.394 (6)	C17—H17	0.9500
C3—H3	0.9500	C18—C19	1.387 (6)
C4—C5	1.378 (6)	C18—H18	0.9500
C4—H4	0.9500	C19—H19	0.9500
C5—C6	1.523 (5)	C20—H20A	0.9800
C6—H6A	0.9900	C20—H20B	0.9800
C6—H6B	0.9900	C20—H20C	0.9800
C7—C8	1.500 (6)	N4—C21	1.160 (5)
C7—H7A	0.9900	C21—S1	1.633 (4)
C7—H7B	0.9900	N5—C22	1.155 (5)
C8—C9	1.397 (6)	S2—C22	1.624 (4)
C9—C10	1.385 (7)		
			
N5—Zn1—N1	108.46 (15)	N3—C8—C9	122.1 (4)
N5—Zn1—N3	123.55 (14)	N3—C8—C7	115.1 (4)
N1—Zn1—N3	123.46 (14)	C9—C8—C7	122.8 (4)
N5—Zn1—N4	101.43 (15)	C10—C9—C8	117.9 (5)
N1—Zn1—N4	99.36 (15)	C10—C9—H9	121.1
N3—Zn1—N4	91.21 (14)	C8—C9—H9	121.1
N5—Zn1—N2	102.49 (13)	C9—C10—C11	119.9 (4)
N1—Zn1—N2	74.73 (12)	C9—C10—H10	120.1
N3—Zn1—N2	73.99 (13)	C11—C10—H10	120.1
N4—Zn1—N2	156.01 (13)	C12—C11—C10	119.0 (5)
C5—N1—C1	118.4 (4)	C12—C11—H11	120.5
C5—N1—Zn1	122.4 (3)	C10—C11—H11	120.5
C1—N1—Zn1	119.1 (3)	N3—C12—C11	121.6 (5)
C6—N2—C7	110.6 (3)	N3—C12—H12	119.2
C6—N2—C13	114.7 (3)	C11—C12—H12	119.2
C7—N2—C13	110.9 (3)	N2—C13—C14	113.1 (3)
C6—N2—Zn1	105.5 (2)	N2—C13—C20	111.9 (3)
C7—N2—Zn1	94.5 (2)	C14—C13—C20	112.6 (3)
C13—N2—Zn1	118.8 (2)	N2—C13—H13	106.2
C8—N3—C12	119.6 (4)	C14—C13—H13	106.2
C8—N3—Zn1	117.1 (3)	C20—C13—H13	106.2
C12—N3—Zn1	123.2 (3)	C19—C14—C15	117.6 (4)
N1—C1—C2	122.4 (4)	C19—C14—C13	119.7 (4)
N1—C1—H1	118.8	C15—C14—C13	122.7 (4)
C2—C1—H1	118.8	C16—C15—C14	120.8 (4)
C1—C2—C3	119.2 (4)	C16—C15—H15	119.6
C1—C2—H2	120.4	C14—C15—H15	119.6
C3—C2—H2	120.4	C17—C16—C15	120.6 (4)
C2—C3—C4	118.9 (4)	C17—C16—H16	119.7
C2—C3—H3	120.5	C15—C16—H16	119.7
C4—C3—H3	120.5	C18—C17—C16	119.5 (4)
C5—C4—C3	118.9 (4)	C18—C17—H17	120.3
C5—C4—H4	120.5	C16—C17—H17	120.3
C3—C4—H4	120.5	C17—C18—C19	119.9 (4)
N1—C5—C4	122.1 (4)	C17—C18—H18	120.0
N1—C5—C6	117.6 (4)	C19—C18—H18	120.0
C4—C5—C6	120.3 (4)	C18—C19—C14	121.6 (4)
N2—C6—C5	112.1 (3)	C18—C19—H19	119.2
N2—C6—H6A	109.2	C14—C19—H19	119.2
C5—C6—H6A	109.2	C13—C20—H20A	109.5
N2—C6—H6B	109.2	C13—C20—H20B	109.5
C5—C6—H6B	109.2	H20A—C20—H20B	109.5
H6A—C6—H6B	107.9	C13—C20—H20C	109.5
N2—C7—C8	109.6 (3)	H20A—C20—H20C	109.5
N2—C7—H7A	109.7	H20B—C20—H20C	109.5
C8—C7—H7A	109.7	C21—N4—Zn1	171.6 (4)
N2—C7—H7B	109.7	N4—C21—S1	179.9 (5)
C8—C7—H7B	109.7	C22—N5—Zn1	170.3 (4)
H7A—C7—H7B	108.2	N5—C22—S2	178.5 (4)
			
C5—N1—C1—C2	0.3 (6)	N3—C8—C9—C10	0.9 (6)
Zn1—N1—C1—C2	−175.7 (4)	C7—C8—C9—C10	179.3 (3)
N1—C1—C2—C3	−1.2 (7)	C8—C9—C10—C11	0.4 (6)
C1—C2—C3—C4	1.4 (7)	C9—C10—C11—C12	−1.3 (6)
C2—C3—C4—C5	−0.8 (6)	C8—N3—C12—C11	0.4 (5)
C1—N1—C5—C4	0.3 (6)	Zn1—N3—C12—C11	−175.8 (3)
Zn1—N1—C5—C4	176.2 (3)	C10—C11—C12—N3	0.9 (7)
C1—N1—C5—C6	−177.4 (4)	C6—N2—C13—C14	70.2 (4)
Zn1—N1—C5—C6	−1.5 (5)	C7—N2—C13—C14	−56.0 (4)
C3—C4—C5—N1	0.0 (6)	Zn1—N2—C13—C14	−163.8 (2)
C3—C4—C5—C6	177.7 (4)	C6—N2—C13—C20	−58.3 (4)
C7—N2—C6—C5	−72.5 (4)	C7—N2—C13—C20	175.5 (3)
C13—N2—C6—C5	161.1 (3)	Zn1—N2—C13—C20	67.7 (4)
Zn1—N2—C6—C5	28.5 (4)	N2—C13—C14—C19	94.8 (4)
N1—C5—C6—N2	−21.4 (5)	C20—C13—C14—C19	−137.1 (4)
C4—C5—C6—N2	160.8 (3)	N2—C13—C14—C15	−85.5 (4)
C6—N2—C7—C8	161.8 (3)	C20—C13—C14—C15	42.7 (5)
C13—N2—C7—C8	−69.7 (4)	C19—C14—C15—C16	−0.1 (6)
Zn1—N2—C7—C8	53.4 (3)	C13—C14—C15—C16	−179.9 (4)
C12—N3—C8—C9	−1.3 (5)	C14—C15—C16—C17	−0.9 (7)
Zn1—N3—C8—C9	175.1 (3)	C15—C16—C17—C18	0.2 (7)
C12—N3—C8—C7	−179.8 (3)	C16—C17—C18—C19	1.5 (6)
Zn1—N3—C8—C7	−3.4 (4)	C17—C18—C19—C14	−2.5 (6)
N2—C7—C8—N3	−41.4 (4)	C15—C14—C19—C18	1.8 (6)
N2—C7—C8—C9	140.1 (4)	C13—C14—C19—C18	−178.4 (3)

### Hydrogen-bond geometry (Å, º)

**Table:**

*D*—H···*A*	*D*—H	H···*A*	*D*···*A*	*D*—H···*A*
C3—H3···S2^i^	0.95	2.77	3.604 (5)	147
C11—H11···S1^ii^	0.95	2.80	3.738 (5)	169

Symmetry codes: (i) −*x*+1, *y*+1, −*z*+2; (ii) −*x*+1, *y*, −*z*+1.
